# What Factors Influence Smoking Prevalence and Smoke Free Policy Enactment across the European Union Member States

**DOI:** 10.1371/journal.pone.0023889

**Published:** 2011-08-31

**Authors:** Ilze Bogdanovica, Ann McNeill, Rachael Murray, John Britton

**Affiliations:** 1 UK Centre for Tobacco Control Studies, University of Nottingham, Nottingham, United Kingdom; 2 Division of Epidemiology and Public Health, University of Nottingham, Nottingham, United Kingdom; University of Texas at Tyler, United States of America

## Abstract

**Background:**

Smoking prevention should be a primary public health priority for all governments, and effective preventive policies have been identified for decades. The heterogeneity of smoking prevalence between European Union (EU) Member States therefore reflects, at least in part, a failure by governments to prioritise public health over tobacco industry or possibly other financial interests, and hence potentially government corruption.

The aims of this study were to test the hypothesis that smoking prevalence is higher in countries with high levels of public sector corruption, and explore the ecological association between smoking prevalence and a range of other national characteristics in current EU Member States.

**Methods:**

Ecological data from 27 EU Member States were used to estimate univariate and multivariate correlations between smoking prevalence and the *Transparency International* Corruption Perceptions Index, and a range of other national characteristics including economic development, social inclusion, quality of life and importance of religion. We also explored the association between the Corruption Perceptions Index and measures of the extent to which smoke-free policies have been enacted and are enforced.

**Results:**

In univariate analysis, smoking prevalence was significantly higher in countries with higher scores for corruption, material deprivation, and gender inequality; and lower in countries with higher *per capita* Gross Domestic Product, social spending, life satisfaction and human development scores. In multivariate analysis, only the corruption perception index was independently related to smoking prevalence. Exposure to tobacco smoke in the workplace was also correlated with corruption, independently from smoking prevalence, but not with the measures of national smoke-free policy implementation.

**Conclusions:**

Corruption appears to be an important risk factor for failure of national tobacco control activity in EU countries, and the extent to which key tobacco control policies have been implemented. Further research is needed to assess the causal relationships involved.

## Introduction

Since cigarette smoking is the leading preventable cause of death, disability and social inequality in health in high and middle income countries [1], [2], smoking prevention should be a major priority for governments of all developed nations. In the European Union (EU) about one in four adults are still regular cigarette smokers and there are marked differences in the level and direction of change in smoking prevalence between Member States. For example, smoking prevalence in Sweden is the lowest in the EU and is still falling, whilst in countries such as Greece, Austria and Bulgaria, prevalence is high and in some cases still rising [3].

Differences in current smoking prevalence between countries in part reflect inevitable differences in stage of progression of the smoking epidemic, but also reflect the extent to which past and current governments have implemented World Health Organisation Framework Convention on Tobacco Control policies [4] to prevent and reverse the progression of the smoking epidemic [5]. However, since most of these policies were first advocated nearly fifty years ago [6], [7], governments, politicians and public health specialists have long been aware that measures such as high taxation, advertising bans, smoke-free legislation and health warnings on cigarette packs are effective in preventing smoking [8]. However, adoption of such policies is a variable and predominantly recent phenomenon in most EU Member States, and remains far from comprehensive [9], [10].

Failure to reduce smoking prevalence may arise either from failure to enact effective tobacco control policies, or from failure to ensure compliance with them. It has previously been reported that smoking prevalence reflects the extent to which effective tobacco control policies are implemented, and that support for and the success of smoke-free policies is greater in EU countries with more advanced tobacco control policies [11]. Since high smoking prevalence therefore reflects health policy failure we hypothesised that higher smoking prevalence would be expected in countries in which health policy was undermined by conflicting interests or cultures, and that in particular, tobacco control policies would be less likely to be implemented or enforced in countries with high levels of corruption. We have therefore studied the association between public sector corruption, defined by *Transparency International* as the abuse of entrusted power for private gain [12], and other national characteristics, and the prevalence of smoking in the current 27 EU Member States.

## Methods

We investigated ecological associations between smoking prevalence in the 27 EU Member States and variables describing various national characteristics identified from existing evidence [13], [14], [15], [16] and internet searches as measures that quantified country characteristics likely to influence smoking prevalence. Data sources identified and used were:

### Smoking prevalence

Smoking prevalence data were taken from the Eurobarometer survey, which measures smoking prevalence in all current 27 EU Member States from samples of around 1,000 respondents (500 in smaller Member States) aged 15 years and older. Since the most recent available data for other country characteristics (below) were available for the years 2007 or 2008, we used 2008 Flash Eurobarometer data for the present analysis [17].

### Corruption

We used the *Transparency International* Corruption Perceptions Index, which measures perceived levels of public sector corruption on a scale from 1 to 10, higher scores representing lower corruption, using data for 2008 [12]. The Corruption Perceptions Index draws on 13 sources provided by 11 independent expert and business institutions which measure different aspects of corruption using strict criteria. The Corruption Perceptions Index is estimated using a two step standardization process as the sources use different scales, to provide a mean value 2008 value which reflects components of data relating to 2008 and 2007 [18].

### National wealth

We measured national wealth as *per capita* Gross Domestic Product (GDP), taking data in Euros from the Eurostat database for the year 2008 (except Romania, for which the most recent data were for 2007) [19].

### Income inequality

We used the ratio of total equivalised disposable income, defined as total household income divided by its age-weighted equivalent size (to take into account the size and composition of household), in the highest relative to the lowest quintiles of income [20], [21], from the Eurostat database for 2008 [22].

### Material deprivation

Material deprivation was measured as the proportion of the population receiving an equivalised income below 60% of the median income, using 2008 data from the Eurostat database (data for the UK and France were provisional) [23].

### Social budget

Data on national spending on social benefits (transfers in cash and in kind to households and individuals, other social protection spending and administration costs) in purchasing power standards (PPS) were obtained from the Eurostat database for 2007 (values for Germany, Spain, France, Italy, Cyprus, Latvia, Lithuania, the Netherlands, Slovenia, Slovakia, Sweden, UK were provisional) [24].

### Life satisfaction

We used national average life satisfaction scores, measured on a scale from 1 to 10 from least to most satisfied, from the Second European Quality of Life Survey for 2007 [25].

### Human development

The Human Development index is a composite index of national human development which combines data on life expectancy at birth, adult literacy, educational enrolment and *per capita* GDP. We used data for 2007 published in the United Nations Development Programme Human Development Report [26].

### Gender equality

We used the Gender Empowerment Measure, a composite index of gender inequality in economic and political participation, and power over economic resources, provided for 26 Member States (Luxembourg unavailable) by the United Nations for 2006 [26].

### Unemployment

Data on the proportion of the labour force (age 15–74) unemployed in 2008 were obtained from the Eurostat database [27].

### Education

Data on the proportion of the population aged 18–24 with at most lower secondary education (early school leavers) were taken from the Eurostat database for 2008 [28].

### Importance of religion

Data on the proportion of respondents in each country reporting that religion is among three of their most important personal values were obtained from the Standard Eurobarometer survey for 2008 [29].

### Tobacco production

We used data on total quantity of raw tobacco delivered by Member States in the year 2008 provided by European Commission Directorate General for Agriculture and Rural Development [30].

### Proportion of ex-smokers

Data on the proportion of people who used to smoke but have stopped were included as a proxy indicator of the current stage of smoking epidemic [5]. We used data for the year 2008 from Eurobarometer survey [17].

We also assessed the extent of overall national tobacco control policy enactment in individual Member States using the Joossens and Raw Tobacco Control Scale (TCS) for 2007 (max 100), and as a specific example of implementation of a currently topical policy we used smoke-free policy TCS scores for smoke free work and other public places (maximum score 22) [9]. Scores for smoke free- policies were given separately for workplaces excluding cafes and restaurants (max 10 points), cafes and restaurants (max 8 points), and public transport and other public places (max 4 points). We also stratified the 27 EU Member States into two groups; those with a high level of smoke free policy implementation and those with low level implementation, using the median value as a cut-off point, and investigated whether association between smoking prevalence and variables that appeared to be significant at univariate level for all countries remained consistent.

We measured enforcement of smoke-free policy using 2008 Flash Eurobarometer survey [17] self-report estimates of the proportion of people exposed to tobacco smoke in the workplace among those working away from home (including any exposure time), and the proportion of indoor workers who do not have any smoking restrictions at their workplace.

### Statistical analysis

We used SPSS v.17 to estimate univariate Spearman Rank correlations, and partial correlation and multiple regression with backwards exclusion to identify associations with smoking prevalence that were independently significant at p<0.05.

## Results

### Correlates of smoking prevalence

Mean and standard deviation values, ranges and countries at the extremes of the ranges for the variables studied are summarised in Table 1. EU Member States involved in tobacco production in 2008 comprised Belgium, Bulgaria, Germany, Greece, Spain, France, Italy, Hungary, Poland, Portugal, and Romania. Average annual tobacco production (including all 8 groups of variety- flue cured, light air cured, dark air cured, fire cured, sun cured, Basmas, Katerini, Kaba Koulak) was 23.417 (SD 27.129) tonnes, ranging from 131 tonnes in Belgium to 92.556 tonnes in Italy.

**Table 1:** Summary of variables.

Variable	Mean (SD)	Range	
		Minimum (Country)	Maximum (Country)
Smoking prevalence (%)	31.4 (4.8)	22.6 (SI)	42.1(EL)
Per capita GDP (Euros)	24,293 (15,923)	4,500 (BG)	80,500 (LU)
Corruption Perceptions Index	6.5 (1.7)	3.6 (BG)	9.3 (DK)
Income inequality	4.7 (1.2)	3.4 (CZ)	7.3 (LV)
Material deprivation (%)	42.2(19.4)	14.1 (SE)	92.8 (BG)
Social budget (PPS* per capita)	5,615.0 (3,064.5)	1352.2 (RO)	13,231.3 (LU)
Life satisfaction	7.0 (0.8)	5.0 (BG)	8.5 (DK)
Human development	0.921 (0.041)	0.837 (BG)	0.965 (IE)
Gender inequality	0.700 (0.121)	0.497 (RO)	0.906 (SE)
Unemployment rate (%)	6.2 (1.9)	2.8 (NL)	11.3 (ES)
Education (Early school leavers, %)	14.3 (8.5)	5.0 (PL)	39.0 (MT)
Religion as personal value (%)	8.3 (6.9)	2.0 (PT)	27.0 (CY)
Proportion of ex-smokers	20.9 (4.2)	12.7(CY)	29.2(NL)
Overall Tobacco Control Scale scores	50.7 (12.8)	35.0 (AT)	93.0 (UK)
Tobacco Control Scale scores for smoke free public places	10.5 (5.2)	2.0 (DE)	21.0 (IE)
Proportion of people who work away from home exposed to tobacco smoke in the workplace (%)	22.59 (11.93)	8.0 (SE)	60.0 (EL)
Proportion of indoor workers with no smoking restriction in the workplace (%)	10.8 (7.78)	3.0 (UK)	38.0 (EL)

*PPS- purchasing power standards.

**Figure 1:**
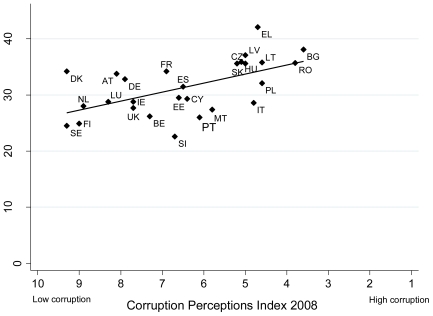
Smoking prevalence and Corruption Perceptions Index score (2008 data).

Smoking prevalence was significantly correlated with the Corruption Perceptions Index (R = −0.583; p = 0.001), *per capita* GDP (R = −0.508; p = 0.007), material deprivation (R = 0.631; p<0.01), social budget (R = −0.509; p = 0.007), life satisfaction (R = −0.624; p = 0.001), human development (R = −0.533; p = 0.004), gender inequality (R = −0.416; p = 0.034), and the proportion of people who used to smoke but have stopped (R = −0.489; p = 0.01) indicating that smoking prevalence tends to be higher in countries with lower national incomes, higher levels of public sector corruption and material deprivation, lower social protection expenditure, lower levels of life satisfaction and human development, and higher levels of gender inequality, but lower levels of proportion of ex-smokers. There was no significant correlation between smoking prevalence and income inequality (R = 0.32; p = 0.103), unemployment (R = 0.190; p = 0.341), educational level (R = −0.012; p = 0.954), importance of religion (R = 0.221; p = 0.268) or tobacco growing (R = 0.164; p = 0.631). Correlations between these variables are shown in Table S1. In a multiple linear regression model with backwards exclusion, starting with all variables significant in univariate analysis, smoking prevalence was independently significantly associated only with the Corruption Perceptions Index score (data shown in Figure 1; prevalence decreasing by 1.62 (95% CI 0.63 to 2.61) per unit on the Corruption Perceptions Index score, p = 0.002). The Corruption Perceptions Index score accounted for 29.5% of the variance of smoking prevalence. Results were similar when alternative modelling technique was used searching for the model explaining most of the variance in smoking prevalence. We also found evidence for some but not high levels of multicollinearity.

To explore the possibility that this finding might differ between the EU countries that became Member States before 2004 (old EU countries- Belgium (BE), Denmark (DK), Germany (DE), Ireland (IE), Greece (EL), Spain (ES), France (FR), Italy (IT), Luxembourg (LU), the Netherlands (NL), Austria (AT), Portugal (PT), Finland (FI), Sweden (SE), the United Kingdom (UK)) and those that joined in 2004 and 2007 (new EU countries- Czech Republic (CZ), Estonia (EE), Cyprus (CY), Latvia (LV), Lithuania (LT), Hungary (HU), Malta (MT), Poland (PL), Slovenia (SI), Slovakia (SK), Romania (RO) and Bulgaria (BG)) we ran the backward regression analysis separately in these groups of countries. In the new EU Member States Corruption Perceptions Index was the only independently significant predictor of smoking prevalence (p = 0.001), and accounted for 63% of the variance in smoking prevalence. In old EU countries, the last variable retained was Gender Empowerment (p = 0.078). Since the Human Development Index is a composite measure that includes components of GDP and educational enrolment, measures of which were also included in our analysis as independent variables (in the form of *per capita* GDP and early school leavers), we repeated the multiple regression excluding the Human Development Index; in this model, the Corruption Perceptions Index and Material Deprivation were the last two variables retained in the model with Material Deprivation being the significant correlate. When we explored regression analysis separately for countries with low and high levels of smoke free policy implementation we found that the final regression model explaining smoking prevalence included Corruption Perceptions Index and *per capita* GDP in countries with low level of smoke free policy but in countries with high levels of smoke free policies the only independently significant predictor of smoking prevalence was life satisfaction.

### Corruption, TCS scores and smoke-free policy enactment and implementation

TCS scores were significantly inversely correlated with smoking prevalence (R = −0.41; p = 0.034) suggesting that smoking prevalence tends to be lower in countries with more comprehensive tobacco control policies in place. Analysis of scores for smoke-free policy revealed that these were significantly and inversely correlated with the proportion of the population reporting no smoking restrictions at work (R = −0.411; p = 0.033), but were not significantly correlated with the proportion reporting exposure to tobacco smoke in the workplace (R = −0.255; p = 0.198). Corruption Perceptions Index scores were unrelated to overall TCS scores (R = 0.130; p = 0.57) or TCS scores for the existence of smoke-free policy (R = −0.027; p = 0.892), but were strongly correlated with the prevalence of workplace exposure (R = −0.769; p<0.01) and an absence of smoking restrictions in the workplace (R = −0.454; p = 0.017). The correlation between the Corruption Perceptions Index and workplace exposure remained significant (R = −0.451; p = 0.021) after controlling for the effect of smoking prevalence. TCS scores for smoke-free policy were also not significantly correlated with any other country characteristic variables (Table S1), or with smoking prevalence (R = −0.311; p = 0.115). We also investigated the consistency of the relation between corruption and enforcement of smoke free policy using data from the 2009 Eurobarometer survey and found similar results indicating borderline significant relationship in the same direction as reported in this study (data not presented). Repetition of this analysis in old and new EU Member States did not reveal any marked differences between them.

## Discussion

Smoking prevalence, and the extent to which policies to prevent smoking have been implemented, varies substantially across the EU [9], [17]. This is the first study to explore the role of country characteristics, and in particular, perceived public sector corruption in determining smoking prevalence and the extent to which smoke free policies are implemented and observed. We demonstrate that smoking prevalence tends to be higher in countries with generally lower levels of income and wellbeing on a range of different measures, but particularly in countries with higher levels of perceived public sector corruption. This association appears to be particularly marked among the newer EU Member States. We also found that whilst the enactment of policies to prevent exposure to tobacco smoke in the workplace was no less likely in relatively corrupt countries, exposure to smoke in the workplace was greater, suggesting a failure to implement or adhere to smoke-free regulations.

Our findings are based on cross-sectional ecological analyses and therefore need to be interpreted with caution, particularly in relation to any causal inference. The data we used were all collected at a time in which EU countries were entering a substantial economic recession, and in absence of more detail and more frequent observations, we are unable to determine whether these unusually stringent economic times influenced our findings. We were prevented from carrying out a more robust analysis of the longitudinal relation between corruption and smoking prevalence by the fact that the methods and sources used to construct the Corruption Perceptions Index vary from year to year, and are therefore not directly comparable over time [31]. The same problem prevented us from analysing prevalence estimates based on national surveys, which as we have previously shown may be more valid estimates of prevalence than those from the small sample sizes used in Eurobarometer, but which are available in only a minority of EU Member States in any one year [32]. However, we conducted the same analyses on smoke free policy implementation using data from other sources (Eurobarometer 2009) and obtained very similar results to those shown here. We chose to analyse smoking prevalence rather than cigarette consumption data because prevalence is the stronger determinant of population health burden, and because relevant data were more readily available. However, it would be useful to investigate whether corruption and other country characteristics are related to cigarette per capita sales data in a similar way. The Corruption Perceptions Index is only one of several measures of corruption, but its major strength is that it combines data from various sources into one index. The Index is primarily focused on views of business people and country analysts, and is designed to provide a cross-sectional rather than longitudinal assessment of corruption levels [31]. However a validation study has reported that levels of perceived corruption obtained using various measures correlate strongly with the Corruption Perceptions Index, making it a valid estimate of perceived corruption [33].

The heterogeneity of smoking prevalence between countries arises in part from their being at different stages of smoking epidemic [5], which in turn reflects differences in social and economic development. However the progression of the epidemic is also determined by the extent to which comprehensive tobacco control policies have been implemented. Smoking is also more prevalent in socioeconomically deprived populations and people with lower levels of education and income [34], and exacerbates deprivation and inequality [14]. Not only wealth but other country characteristics, for example, corruption, might influence success in tobacco control. Whilst corruption itself contributes to poverty [35] and is inversely correlated with GDP, and poorer countries in the EU tend to be at an earlier stage of the smoking epidemic [36], it is also plausible that strong commercial interests such as the tobacco industry are likely to thrive in corrupt environments in which tobacco control measures can more easily be delayed or devalued [37], [38]. However, in our study corruption remained significantly correlated with smoking prevalence even after allowing for GDP.

Our primary objective in this study was to determine whether corruption predicts smoking prevalence; our secondary aim was to provide some insight into the likely mechanism. Although we were only able to identify one tobacco control measure for which suitable data were available out of many that would be interesting to study, our findings for smoke-free policy indicate that the effect of corruption is not to inhibit the passage of measure to restrict smoking, but instead to reduce the extent to which these measures are observed and indeed enforced. One inference that can be drawn from this is that corrupt governments are willing to act to be seen to do the right things for health, but then choose or neglect to ensure that those measures are observed. In addition, it may be that populations in corrupt countries are, for many potential reasons, less likely to feel obliged to observe public health measures. Data from analysis stratifying countries as with high and low levels of smoke free policy implementation confirmed that the univariate association between prevalence of smoking and corruption was true independently of level of smoke free policy implementation. However multivariate analysis suggested that corruption is a significant predictor of smoking prevalence only in countries with low level of smoke free policy implementation. On the data available to us we were unable to study the implementation of other tobacco control policies in a similar way, though the World Bank has reported that in countries with higher corruption, tobacco smuggling is more common [39]. We acknowledge that our analysis is cross-sectional, and that analysis of the relation between longitudinal trends in these variables, when possible, is likely to be more informative of any causal relation between them.

Tobacco companies have a vested interest in and a history of inhibiting both enactment of and compliance with tobacco control policies [40], and Article 5.3. of the World Health Organization's Framework Convention on Tobacco Control [4], which is approved by the European Council and ratified by almost all EU countries, suggests that tobacco control policies should be protected from commercial interests. However, when decisions on tobacco control are made, economic interests are affected [41] and financial or other incentives to defer or dilute policy may well come into play. These need not involve direct individual financial gain; the financial benefit might arise from donations to political parties, or provision of benefits in kind [42], [43]. However our study suggests that strong governance is important in preventing tobacco smoking, and strong and transparent political leadership has the key role in ensuring that effective tobacco control policies are both implemented and observed in the EU.

## Supporting Information

Table S1 Correlations between variables. *Only 11 countries included.
(DOC)
