# Supplementary material for: What Factors Influence Smoking Prevalence and Smoke Free Policy Enactment across the European Union Member States
Source: PLoS One. 2011 Aug 31;6(8):e23889. doi: 10.1371/journal.pone.0023889 (PMC3166128; doi:10.1371/journal.pone.0023889)
Supplement: Table S1 — Correlations between variables. *Only 11 countries included. (DOC) [file pone.0023889.s001.doc]

|  | Smoking prevalence | *Per capita* GDP (Euros) | Corruption Perceptions Index | Income inequality | Material deprivation (%) | Social budget (PPS* per capita) | Life satisfaction | Human development | Gender inequality | Unemployment rate (%) | Education (Early school leavers, %) | Religion as personal value (%) | Tobacco production* | Proportion of ex-smokers | Overall TCS scores | TCS scores for smoke free policy | Daily workplace exposure | No smoking restrictions at work |
| --- | --- | --- | --- | --- | --- | --- | --- | --- | --- | --- | --- | --- | --- | --- | --- | --- | --- | --- |
| Smoking prevalence | 1.00 |  |  |  |  |  |  |  |  |  |  |  |  |  |  |  |  |  |
| *Per capita* GDP (Euros) | -0.508 (0.007) | 1.00 |  |  |  |  |  |  |  |  |  |  |  |  |  |  |  |  |
| Corruption Perceptions Index | -0.583 (0.001) | 0.894 (<0.01) | 1.00 |  |  |  |  |  |  |  |  |  |  |  |  |  |  |  |
| Income inequality | 0.320 (0.103) | -0.411 (0.033) | -0.516 (<0.01) | 1.00 |  |  |  |  |  |  |  |  |  |  |  |  |  |  |
| Material deprivation (%) | 0.631 (<0.01) | -0.812 (<0.01) | 0.807(<0.01) | 0.307 (0.119) | 1.00 |  |  |  |  |  |  |  |  |  |  |  |  |  |
| Social budget (PPS* per capita) | -0.509 (0.007) | 0.946 (<0.01) | 0.848 (<0.01) | -0.412 (0.033) | -0.758 (<0.01) | 1.00 |  |  |  |  |  |  |  |  |  |  |  |  |
| Life satisfaction | -0.624 (0.001) | 0.819 (<0.01) | 0.835 (<0.01) | -0.482 (0.011) | -0.875 (<0.01) | 0.759 (<0.01) | 1.00 |  |  |  |  |  |  |  |  |  |  |  |
| Human development | -0.533 (0.004) | 0.950 (<0.01) | 0.828 (<0.01) | -0.367 (0.060) | -0.820 (<0.01) | 0.914 (<0.01) | 0.806 (<0.01) | 1.00 |  |  |  |  |  |  |  |  |  |  |
| Gender inequality | -0.416 (0.034) | 0.821 (<0.01) | 0.786 (<0.01) | -0.227 (0.264) | -0.725 (<0.01) | 0.826 (<0.01) | 0.653 (<0.01) | 0.808 (<0.01) | 1.00 |  |  |  |  |  |  |  |  |  |
| Unemployment rate (%) | 0.190 (0.341) | -0.250 (0.208) | -0.328 (0.095) | 0.273 (0.168) | 0.222 (0.265) | -0.193 (0.335) | -0.273 (0.168) | -0.134 (0.505) | -0.041 (0.841) | 1.00 |  |  |  |  |  |  |  |  |
| Education (Early school leavers, %) | -0.012 (0.954) | -0.118 (0.557) | -0.205 (0.304) | 0.613 (<0.01) | -0.103 (0.609) | -0.120 (0.550) | -0.181 (0.366) | -0.314 (0.506) | -0.163 (0.426) | 0.212 (0.289) | 1.00 |  |  |  |  |  |  |  |
| Religion as a personal value (%) | 0.221 (0.268) | -0.299 (0.130) | -0.386 (0.047) | 0.195 (0.330) | 0.341 (0.082) | -0.347 (0.076) | -0.105 (0.602) | -0.308 (0.118) | -0.438 (0.025) | 0.018 (0.928) | 0.033 (0.871) | 1.00 |  |  |  |  |  |  |
| Tobacco production* | 0.164 (0.631) | -0.237 (0.483) | -0.427 (0.190) | 0.105 (0.759) | -0.073 (0.832) | -0.173 (0.612) | -0.137 (0.688) | 0.064 (0.853) | -0.145 (0.670) | -0.041 (0.905) | -0.037 (0.915) | 0.064 (0.851) | 1.00 |  |  |  |  |  |
| Proportion of ex-smokers | -0.489 (0.01) | 0.650 (<0.01) | 0.743 (<0.01) | -0.377 (0.053) | -0.696 (<0.01) | 0.645 (<0.01) | 0.744 (<0.01) | 0.684 (<0.01) | 0.690 (<0.01) | -0.206 (0.302) | -0.310 (0.115) | -0.180 (0.369) | 0.146 (0.669) | 1.00 |  |  |  |  |
| Overall TCS scores | -0.410 (0.034) | 0.117 (0.561) | 0.130 (0.570) | 0.064 (0.753) | -0.380 (0.050) | 0.060 (0.765) | 0.387 (0.046) | 0.243 (0.222) | 0.233 (0.252) | 0.122 (0.546) | 0.219 (0.273) | 0.188 (0.348) | 0.146 (0.669) | 0.369 (0.058) | 1.00 |  |  |  |
| TCS scores for smoke free policy | -0.311 (0.115) | 0.050 (0.083) | -0.027 (0.892) | 0.246 (0.216) | -0.363 (0.063) | 0.005 (0.979) | 0.253 (0.204) | 0.184 (0.357) | 0.135 (0.511) | 0.183 (0.361) | 0.241 (0.226) | 0.192 (0.338) | 0.434(0.183) | 0.211 (0.292) | 0.782 (<0.01) | 1.00 |  |  |
| Daily workplace exposure | 0.754 (<0.01) | -0.699 (<0.01) | -0.769 (<0.01) | 0.281 (0.155) | 0.709 (<0.01) | -0.699 (<0.01) | -0.612 (<0.01) | -0.712 (<0.01) | -0.706 (<0.01) | 0.073 (0.719) | 0.050 (0.806) | 0.533 (<0.01) | 0.342 (0.304) | -0.614 (<0.01) | -0.327 (0.096) | -0.255 (0.198) | 1.00 |  |
| No smoking restrictions at work | 0.605 (0.001) | -0.501 (<0.01) | -0.567 (<0.01) | 0.168 (0.403) | 0.656 (<0.01) | -0.493 (0.009) | -0.576 (0.002) | -0.504 (0.007) | -0.458 (0.019) | 0.113 (0.576) | -0.032 (0.873) | 0.332 (0.091) | 0.06 (0.861) | -0.583 (0.01) | -0.480 (0.011) | -0.411 (0.033) | 0.799 (<0.01) | 1.00 |
